# Population-level mortality burden from novel coronavirus (COVID-19) in Europe and North America

**DOI:** 10.1186/s41118-021-00115-9

**Published:** 2021-04-16

**Authors:** Samir Soneji, Hiram Beltrán-Sánchez, Jae Won Yang, Caroline Mann

**Affiliations:** 1 University of North Carolina, Chapel Hill, Rosenau Hall, 135 Dauer Drive, Chapel Hill, NC 27599 USA; 2 University of California, Los Angeles, California USA; 3 Brown University, Providence, Rhode Island USA

**Keywords:** COVID-19, Age-standardized death rate, Infectious disease

## Abstract

As of 31 January 2021, 63.9 million cases and 1.4 million deaths had been reported in Europe and North America, which accounted for 62.5% and 62.4% of the global total, respectively. Comparing the level of mortality across countries has proven difficult because of inherent limitations in the most commonly cited measures (e.g., case-fatality rates). We collected the cumulative number of confirmed deaths from COVID-19 by age in 2020 from the L’Institut National d’études Démographiques (INED) database and Statistics Canada for 15 European and North American countries. We calculated age-specific death rates and age-standardized death rates (ASDR) for each country over a 1-year period from 6 February 2020 (date of first COVID-19 death in Europe and North America) to 5 February 2021 using established demographic methods. We estimated that COVID-19 was the second leading cause of death behind cancer in England and Wales and France and the third leading cause of death behind cancer and heart disease in nine countries including the US. Countries with higher all-cause mortality prior to the COVID-19 experienced higher COVID-19 mortality than countries with lower all-cause mortality prior to the pandemic. The COVID-19 ASDR varied substantially within country (e.g., a 5-fold difference among the highest and lowest mortality states in Germany). Consistently strong public health measures may have lessened the level of mortality for some European and North American countries. In contrast, many of the largest countries and economies in these regions may continue to experience a high mortality level because of poor implementation and adherence to such measures.

**Supplementary Information:**

The online version contains supplementary material available at 10.1186/s41118-021-00115-9.

## Introduction

The first confirmed case of the novel coronavirus (COVID-19) in Europe and North America occurred in January 2020 (Coronavirus disease 2019 (COVID-19) situation report - 5, 2020; Coronavirus disease 2019 (COVID-19) situation report - 27, 2020). The first confirmed deaths from COVID-19 occurred soon thereafter in February 2020. As of 31 January 2021, 63.9 million cases and 1.4 million deaths had been reported in Europe and North America, which accounted for 62.5% and 62.4% of the global total, respectively (Coronavirus disease (COVID-19): Global epidemiological situation, 2020). Over the course of the pandemic, public health containment and mitigation measures, including testing, have varied widely in Europe and North America (All state comparison of testing efforts, n.d.; State COVID-19 Dashboard, 2020). These measures have also varied within individual countries and likely contributed to regional differences in the level of COVID-19 mortality (Chiu, Fischer, & Ndeffo-Mbah, 2020; Nasr, 2020).

Mortality from COVID-19 has been substantial in Europe and North America and may rival the leading causes of death. However, comparing the level of mortality among countries has proven difficult because of inherent limitations in the most commonly cited measures. The case fatality rate, for example, is computed as the ratio of deaths due to COVID-19 to the number of people infected by the disease. However, international comparison of case fatality rates is problematic because COVID-19 is more fatal among the elderly and age distributions differ across countries (COVID-19 hospitalization and death by age, 2020). Additionally, the *true* number of cases may far exceed the reported number of cases (i.e., the denominator of the case fatality rate) in most countries due to a lack of widespread testing (Thomas, 2020).

We addressed this research gap and compared the level of COVID-19 mortality across countries by calculating age-specific death rates based on the number of confirmed deaths. In contrast to case fatality rates, age-specific death rates equal the ratio of deaths due to COVID-19 to the exposure measured in person-years lived by age. Age-specific death rates can then be summarized into the age-standardized death rate, which accounts for differences in the age distribution of countries and facilitates appropriate international comparison. We also assessed the level of COVID-19 mortality within each country by comparing its crude death rate against the previous leading causes of death in that country. Finally, we assessed within-country variation in the level of COVID-19 mortality (e.g., among states in the US). We made available easy-to-use software for public health officials to assess the level of COVID-19 mortality with updated epidemiologic information.

## Methods

### Data

We collected the cumulative number of confirmed deaths from COVID-19 by age from the L’Institut National d’études Démographiques (INED). European and North American countries in the INED database included: Austria, Belgium, England and Wales, Denmark, France, Germany, Italy, the Netherlands, Norway, Portugal, Scotland, Spain, Sweden, and the United States (US). INED compiles data from each country’s health ministry on a weekly basis. For example, the source of data for France is Santé Publique France (Public Health France). Although Canada was not included in the INED database, the Government of Canada publishes the cumulative number of confirmed deaths from COVID-19 by age directly on its official COVID-19 data dashboard (Coronavirus disease 2019 (COVID-19): Epidemiology update, 2021). We collected age-specific population counts in 2019 for each country (except Canada) from the INED database; age-specific population counts in 2019 for Canada came from the Human Mortality Database. We also collected total population counts in 2020 for each country projected by the United Nations (UN).

We collected death counts by leading causes of death in 2017 (the most recent year for which cause-specific mortality data was available) for each European Union (EU) country from Eurostat, the statistical office of the EU (Causes of Death (Hlth_cdeath), 2020). We collected similar cause-specific death counts for Canada from Statistics Canada, England and Wales from the Office for National Statistics, Scotland from the National Records Scotland, and the US from the National Center for Health Statistics. Finally, we collected all-cause death rates in 2017 for each country from the Human Mortality Database.

### International analysis

First, we estimated the age-specific population counts in 2020 for each country by multiplying [1] the age-specific population counts in 2019 for each country by [2] the ratio of the total population counts in 2020 and 2019 projected by the UN (i.e., we held constant the proportion of the population by age in 2019 for each country). For England and Wales and Scotland, we applied the ratio of the total population counts in 2020 and 2019 for the entire UK projected by the UN.

Second, we calculated the age-specific death rates from COVID-19 for each country, which equaled the ratio of age-specific death counts from COVID-19 to age-specific population counts, over the 1-year period from 6 February 2020 (date of the first reported COVID-19 death in Europe and North America) to 5 February 2021. The width of age groups varied among countries (e.g., 10-year age groups in Germany). The starting age of the final age group also varied among countries: 80 years for 5 countries (Canada, England and Wales, Portugal, Spain, and Switzerland), 85 years for 4 countries (Austria, Belgium, Scotland, and the US), 90 for 6 countries (Denmark, France, Germany, Italy, Norway, and Sweden), and 95 for 1 country (the Netherlands). National health agencies varied in the frequency in which they reported death counts (e.g., daily reporting in France and weekly reporting in the US). Thus, the reporting day closest to 5 February 2021 differed slightly for each country (e.g., 27 January 2021 for the US). We then annualized the age-specific death rates over the 1-year period from 6 February 2020 to 5 February 2021. For example, 358 days of exposure elapsed from the start of the study period (6 February 2020) to the final reporting day for the US (27 January 2021). We annualized the US age-specific death rates over this time period by multiplying them by the ratio of: [1] 365 days in a calendar year and [2] 358 days of exposure.

Third, we calculated the COVID-19 crude death rate (CDR), which equaled the ratio of total COVID-19 death counts (i.e., across all ages) to total population counts. We similarly annualized COVID-19 CDRs as described earlier. For each country, we compared the CDR from COVID-19 with the CDR of the previous leading causes of death. Leading causes of death included Alzheimer’s disease, cancer, cerebrovascular disease, chronic lower respiratory disease, diabetes mellitus, diseases of the kidney and ureter, heart disease, influenza and pneumonia, intentional self-harm, and unintentional injury accidents.

Fourth, we calculated the age-standardized death rate (ASDR) from COVID-19 for each country. The ASDR equals the weighted average of the annualized COVID-19 age-specific death rates, where the weights reflected the population structure of the 2013 European Standard Population (Revised European Standard Population 2013 (2013 ESP), 2013). Fourth, we also estimated the all-cause ASDR in 2017 for each country utilizing all-cause death rates from period life tables and same European Population Standard weights. We then fit a weighted linear regression on the COVID-19 ASDR in 2020 as a function of all-cause ASDR in 2017 to assess the relationship between population health before the pandemic and during the pandemic. The weights reflected the total population size of each country.

### Within-country analysis

We considered the case of Germany and the US—the two largest countries by population in Europe and North America, respectively—to assess within-country variation in the COVID-19 ASDR. We collected the cumulative number of confirmed deaths from COVID-19 by age and länder (Germany) and state (the US) from the Robert Koch Institute (Germany) and the Centers for Disease Control and Prevention (the US). Second, for Germany, we collected projected population counts in 2020 by age group and länder from Statistisches Bundesamt. For the US, we collected population counts by age derived from the 2018 American Community Survey and published by the Census Bureau and annual population projections published by the UN (Age and Sex, 2018; World Population Prospects 2019, 2019). We calculated the distribution of the US population by age group and state in 2018. We then prorated the UN projection of the total US population in mid-2020 across state and age group by holding constant the age-state proportions estimated for the 2018 population. We then calculated the COVID-19 ASDR following the steps described earlier for the 50 states and the District of Columbia in the US and the 16 länder in Germany. The age standard for calculation of the ASDR was based on the national population counts in 2010 for each country published by the Human Mortality Database.

## Results

### International analysis

**Fig. 1:**
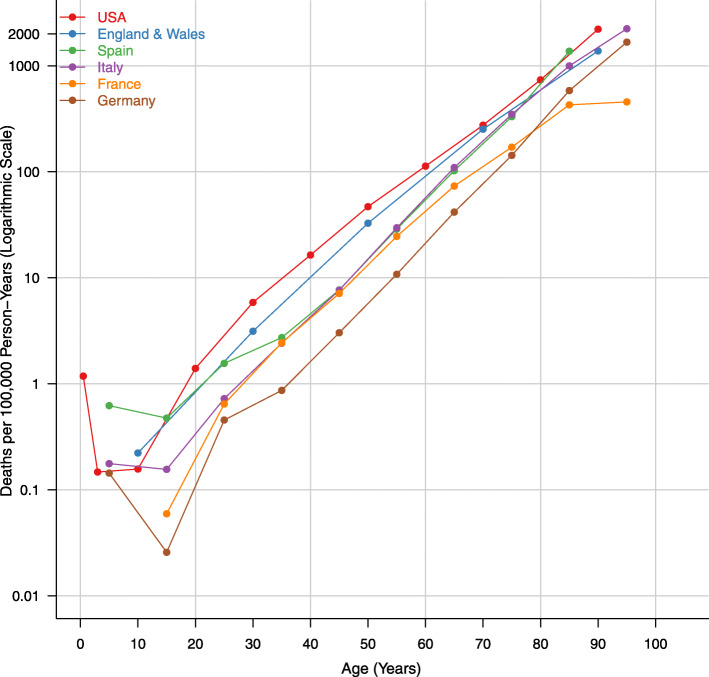
Age-specific death rates from COVID-19 in England and Wales, France, Germany, Italy, Spain, and the US. Source: INED

Among the six largest countries in our analysis (US, Germany, France, Italy, England and Wales, and Spain), the COVID-19 death rate increased exponentially with age and varied across countries within age groups (Fig. 1). For example, the death rate among 55–64 year olds in the US was 113.0 deaths per 100,000 person-years compared to 41.7 deaths per 100,000 person-years among 60–69 year olds in Germany. We observed similar patterns among the other European countries and Canada (Additional file 1: Figure 1). For example, the death rate among 80–89 year olds in Sweden was 1189.5 deaths per 100,000 person-years compared to 111.0 deaths per 100,000 person-years for the same age group in Norway.

As a result of differences in age-specific death rates, the COVID-19 CDR varied across the US, Canada, and European countries (Table 1). It exceeded 100 deaths per 100,000 person-years in Belgium (180.9), England and Wales (124.9), Italy (137.8), Portugal (131.5), Scotland (150.7), Spain (127.7), Sweden (118.8), Switzerland (101.8), and the US (122.7). It fell below 50 deaths per 100,000 person-years in two countries: Norway (10.7) and Denmark (37.6). Compared to previous leading causes of death, COVID-19 was the second leading cause of death behind cancer in 2020 in two countries: England and Wales and France. COVID-19 was the third leading cause of death behind cancer and heart disease in eleven of the sixteen countries: Austria, Belgium, Germany, Italy, the Netherlands, Portugal, Scotland, Spain, Sweden, Switzerland, and the US. COVID-19 only fell below the 10th leading cause of death in Norway.

**Table 1:** COVID-19 crude death rate and rank among leading causes of death

Country	COVID-19 crude death rate (deaths per 100,000 person-years)	Cause of death rank
England and Wales	124.9	2
France	94.3	2
Belgium	180.9	3
Germany	68.4	3
Italy	137.8	3
Netherlands	81.7	3
Portugal	131.5	3
Scotland	150.7	3
Spain	127.7	3
Sweden	118.8	3
Switzerland	101.8	3
US	122.7	3
Denmark	37.6	5
Canada	50.5	9
Norway	10.7	10

Source: authors’ calculations

**Fig. 2:**
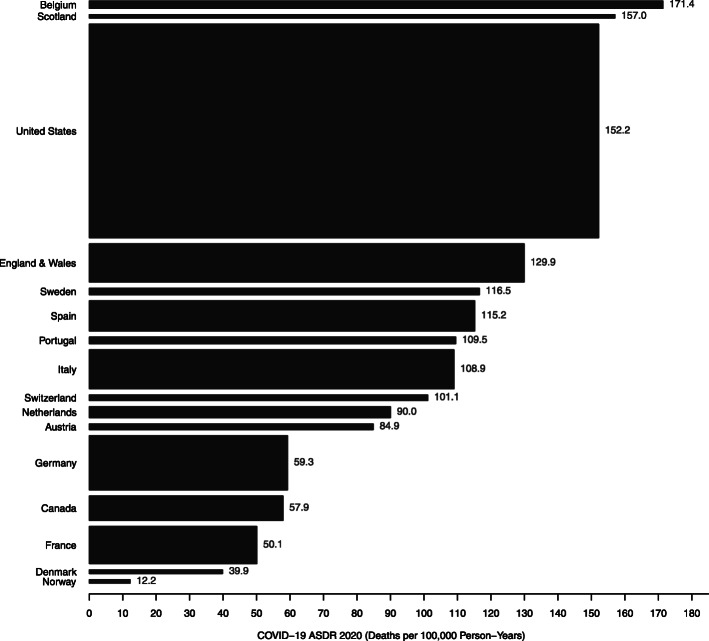
Age-standardized death rate from COVID-19 in Europe and North America. Source: authors’ calculations. Note: the height of each rectangle is proportional to the population size of each country

The ASDR also varied across countries (Fig. 2). It exceeded 100 deaths per 100,000 person-years in Belgium (171.4), Scotland (157.0), the US (152.2), England and Wales (129.9), Spain (115.2), Italy (108.9), and Switzerland (101.1). The COVID-19 ASDR fell below 50 deaths per 100,000 person-years in Norway (12.2) and Denmark (39.9).

**Fig. 3:**
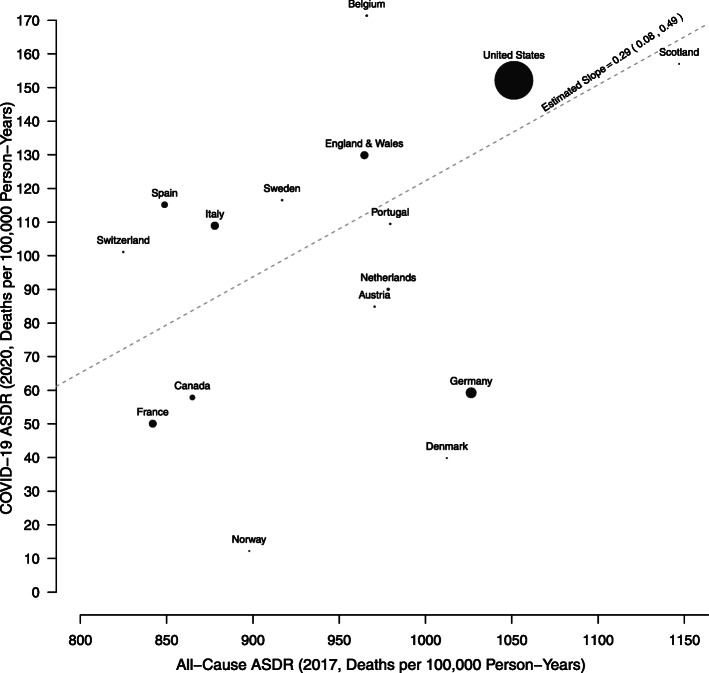
All-cause ASDR in 2017 and COVID-19 ASDR in 2020 in Europe and North America. Source: authors’ calculations. Note: the diameter of each circle is proportional to the population size of each country

We measured the pre-COVID-19 health status of each country by the all-cause ASDR in 2017. We observed a significant relationship between the all-cause ASDR in 2017 and COVID-19 ASDR in 2020 (Fig. 3). In other words, countries with lower all-cause mortality in 2017 also experienced lower COVID-19 mortality in 2020. A notable exception occurred between Sweden and Norway, which experienced nearly the same all-cause ASDR in 2017: 916.9 and 897.9 deaths per 100,000 person-years, respectively. However, the COVID-19 ASDR in 2020 was substantially higher in Sweden (116.5 deaths per 100,000 person-years) than in Norway (12.2 deaths per 100,000 person-years).

### Within-country analysis

**Fig. 4:**
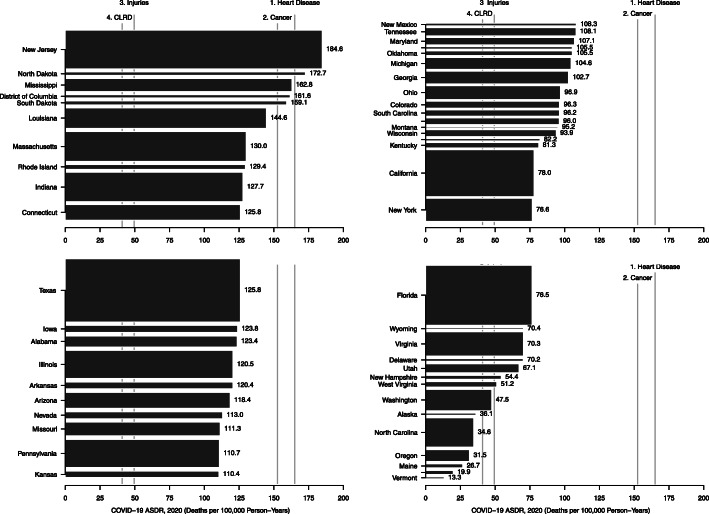
Age-standardized death rate from COVID-19 in the US. Source: authors’ calculations. Note: the height of each rectangle is proportional to the population size of each country

**Fig. 5:**
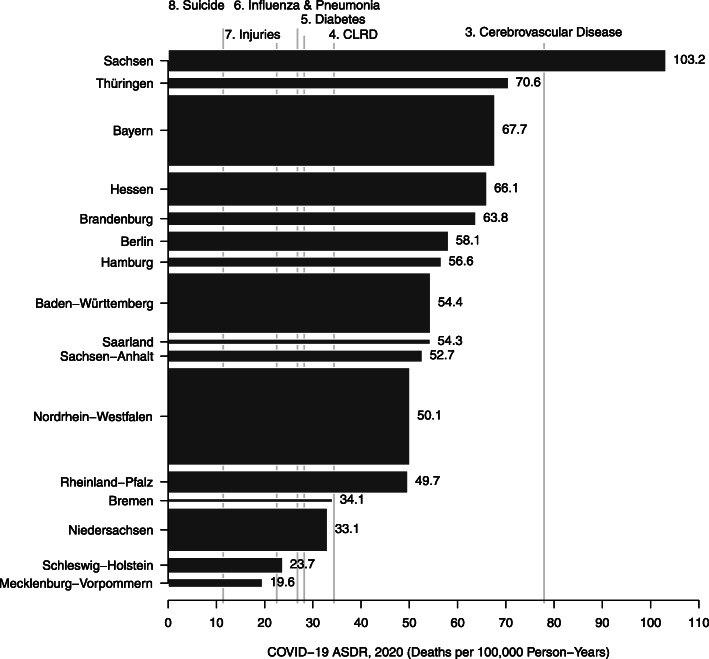
Age-standardized death rate from COVID-19 in Germany. Source: authors’ calculations. Note: the height of each rectangle is proportional to the population size of each country

We observed substantial within-country variation in the COVID-19 ASDR across länder and states in Germany and the US, respectively. In the US, the COVID-19 ASDR varied from 184.6 deaths per 100,000 person-years in New Jersey to 13.3 deaths per 100,000 person-years in Vermont (Fig. 4). In New Jersey, COVID-19 was the leading cause of death exceeding the ASDR of heart disease, the previous leading causes of death nationally. In North Dakota, Mississippi, the District of Columbia, and South Dakota, COVID-19 was the second leading cause of death exceeding the ASDR for cancer, the previous second leading cause of death nationally. In Germany, the COVID-19 ASDR varied from 103.2 deaths per 100,000 person-years in Sachsen to 19.6 deaths per 100,000 person-years in Mecklenburg-Vorpommern (Fig. 5). In Sachsen, COVID-19 was the third leading cause of death exceeding the ASDR of cerebrovascular disease, the previous third leading cause of death nationally. In 12 länder, including Nordrhein-Westfalen, COVID-19 was the fourth leading cause of death exceeding the ASDR of chronic lower respiratory disease, the previous fourth leading cause of death nationally.

## Discussion

This study reports four central findings on the level of COVID-19 mortality in 2020, as measured by its ASDR. First, COVID-19 was the second leading cause of death in 2020 in England and Wales and France and the third leading cause in eleven of the sixteen countries studied, including the US. Second, the COVID-19 ASDR varied considerably across countries: a 9-fold difference between Sweden and Norway and a 2-fold difference between the US and Canada. Third, countries with higher all-cause mortality prior to the pandemic experienced higher COVID-19 mortality than countries with lower all-cause mortality prior to the pandemic. Fourth, the COVID-19 ASDR varied substantially within Germany and the US: a 5-fold and 14-fold difference among the highest and lowest mortality länder and states, respectively.

Most countries in Europe, North America, and elsewhere struggled with developing and implementing adequate public health measures early in the pandemic (Ward, 2020). Italy, for example, first locked-down cities, then regions, and finally the entire country (Horowitz, 2020). However, loopholes reduced the effectiveness of the lockdowns (Horowitz, Bubola, & Povoledo, 2020), and the Italian populace failed to adhere to the lockdowns and stay-at-home orders, partly due to the government’s failure to communicate the seriousness of the pandemic (Horowitz et al., 2020). Similarly, the British government failed to implement effective social distancing measures early in the pandemic (Hunter, 2020). The government tried to prevent overwhelming the capacity of acute care hospitals—as had happened in Italy—by discharging patients to care homes, which had less clinical expertise and medical equipment than hospitals (Booth, 2020). The decision directly contributed to dozens of outbreaks and thousands of COVID-19 deaths within care homes (Booth, 2020). Between 10 April and 15 May 2020, care homes recorded approximately 53% of all COVID-19 deaths that occurred in England (Coronavirus (COVID-19) in the UK: Deaths, 2020).

The substantial difference in the COVID-19 ASDR in Sweden compared to other Scandinavian countries highlights the consequence of markedly different approaches to the pandemic. Norway and Denmark both shut down businesses, closed schools, and mandated face coverings (Han et al., 2020; Vogel, 2020). In contrast, Sweden has kept its economy open and instead pursued a controversial strategy of herd immunity (Habib, 2020). This strategy, which began prior to the development of an efficacious or effective vaccine, may have contributed to an excess number of COVID-19 cases and deaths (25 Swedish doctors and scientists, 2020).

Similarly, in the US, several logistical barriers prevented widespread testing and, consequently, led to a large number of undocumented cases early in the epidemic (Carey, 2020; Li et al., 2020; Madrigal & Meyer, 2020a). First, the US experienced delays in developing and deploying test kits (Fink & Baker, 2020). Second, the US suffered from a supply shortage of materials required for these tests (e.g., reagents to extract genetic material from viruses present in samples) (Lim & Ehley, 2020). Third, unlike other countries, laboratories were not sufficiently organized to analyze the large number of tests in a timely manner (Barone, 2020; Ognyanova, Lazer, & Baum, 2020).

As of late January 2021, the US has conducted 295 million COVID-19 tests, which was more than any other country (How does testing in the U.S. compare to other countries?, 2020; US Historical Data, 2020). However, the ability of testing to reduce COVID-19 deaths depends on more than just the number of tests. The false positive rate of rapid antigen tests may exceed commonly accepted standards (e.g., 15% for the Abbot ID Now test) (Madrigal & Meyer, 2020b; Stein, 2020). Viral tests that detect nucleic acid via reverse transcription polymerase chain reaction are more accurate than antigen tests (Interim guidance for rapid antigen testing for SARS-CoV-2, 2020). Yet, as the demand for testing has increased, so too has the turnaround time as results may take a week or longer (Galewitz & Volz, 2020). Consequently, infected individuals may unknowingly spread the infection for several days while waiting for results before they learn they had tested positive. In an analysis of 181 confirmed cases with known dates of exposure and symptom onset, the incubation period for COVID-19 varied between 2 and 12 days (mean 5 days) (Lauer et al., 2020), which is longer than the 2-day incubation period typically observed with the seasonal flu (Clinical signs and symptoms of influenza, 2020).

In contrast to many European countries and the US, Germany implemented widespread testing and contact tracing early in the epidemic, and the populace adhered to social distancing measures (Beaumont, 2020; Bennhold, 2020). The effective shutdown of businesses and schools helped prevent a surge of hospitalizations such as those experienced in France and Italy (Bennhold, 2020). Despite effective public health measures, the burden of COVID-19 was still high among residents of German care centers; approximately one-third of all COVID-19 deaths have occurred among residents of these facilities (Birnbaum & Booth, 2020).

Public health experts and government officials warned of an impending second wave of cases and deaths as the 2020–2021 winter season approached, which reduces the opportunity for people to physically distance outside (Landler, 2020; Mallapaty, 2020). The US, for example, experienced a peak level of daily incident cases (nearly 250,000) in mid-January 2021 (CDC COVID data tracker, 2021). Widespread lockdowns could help reduce the volume of cases, but such measures are politically challenging and come at severe economic costs (Adam, 2020). Greater epidemiological knowledge of COVID-19 may provide additional tools for governments. The dispersion factor, *k*, of COVID-19 may be lower than other infectious diseases (Kupferschmidt, 2020a). A recent analysis of COVID-19 transmission found that about 80% of secondary transmissions were caused by about 10% of infected individuals (compared to approximately 16% for severe acute respiratory syndrome [SARS] and 25% for Middle East respiratory syndrome [MERS]) (Endo et al., 2020). Thus, governments may be able to reduce transmission by limiting so-called super-spreader events and implementing effective contact tracing focused on identifying the source (i.e., person) of the transmission (Kupferschmidt, 2020b). Public health measures to reduce transmission may be even more salient given newer and more virulent strains of COVID-19. For example, surveillance studies suggest that the B.1.1.7 variant (the UK COVID strain) may be 30 to 70% more transmissible than the original variant and possibly more lethal, too (Booth, 2021; New COVID-19 variants, 2021).

Inadequate implementation of and adherence to public health measures by state governments may have contributed to the substantial within-country variation in the COVID-19 ASDR observed in Germany and the US. The governors of Sachsen and Thüringen in Germany, where the COVID-19 ASDR was highest, now acknowledge that poor government planning in the fall of 2020 contributed to the wave of COVID-19 cases among the elderly in the winter of 2020–2021 (Nasr, 2021). Similarly, public health measures differ substantially among US states. As of early 2021, 13 states have lifted previous bans on large gatherings, 12 states have not issued state-wide requirements for face coverings among the general public, and testing rates per capita varied 5-fold among states (All state comparison of testing efforts, n.d.; State COVID-19 Dashboard, 2020).

We note several limitations. First, the number of COVID-19 deaths in several countries may be higher than officially reported, especially among the elderly, due to lack of testing (Laurent, 2020; Parodi, 2020; Stancati & Sylvers, 2020; Uncounted, unseen - Many covid deaths in care homes are unrecorded, 2020). Individuals who died at home or in a nursing home or care home may not have been tested for COVID-19 as testing was reserved for those hospitalized with severe symptoms. The deaths of those who died without having been tested may not have been attributed to COVID-19 (Walsh & Krever, 2020). Thus, our calculation of the ASDR may serve as a conservative estimate as it was based on reported deaths. Additionally, countries define COVID-19 deaths differently (Coronavirus disease 2019 (COVID-19) | 2020 interim case definition, 2020; Cross-country analysis, 2020). Some countries (e.g., France, Germany, Canada, and the US) apply the World Health Organization definition based on clinically confirmed or probable COVID-19 cases; the WHO definition does not require confirmation of COVID-19 infection from a laboratory test. Other countries require laboratory confirmation (e.g., Sweden and Norway). Thus, our calculation of the ASDR may also serve as a conservative estimate in these countries because some COVID-19 deaths may not have been confirmed by a positive test result (either because of limited testing or a false negative test result). Second, we excluded other European and North American countries because they were not in the INED database and did not publish COVID-19 deaths by age on official government websites (e.g., COVID-19 dashboards).

In conclusion, the level of COVID-19 mortality has varied and will likely continue to vary substantially across Europe and North America. Early and successful containment and mitigation measures may have lessened the mortality level for some countries. However, many of the largest countries and economies may experience a high burden including England and Wales, France, Italy, and the US. Assessing the level of COVID-19 mortality within and among countries requires the use of appropriate measures, such as the age-standardized death rate. Continual assessment will help governments quantify the effect of current and new public health measures as the pandemic continues.

## Supplementary Information

**Additional file 1: Figure 1.**

## Data Availability

The datasets generated and analyzed during the current study are available in the Harvard Dataverse repository, 10.7910/DVN/6DXCRO.

## Abbreviations

COVID-19: 2019 novel coronavirus
US: The United States of America
ASDR: Age-standardized death rates
INED: L’Institut National d’études Démographiques
EU: European Union
CDR: Crude death rate
