# Supplementary figures and images for: Population-level mortality burden from novel coronavirus (COVID-19) in Europe and North America
Source: Genus. 2021 Apr 16;77(1):7. doi: 10.1186/s41118-021-00115-9 (PMC8050994; doi:10.1186/s41118-021-00115-9)

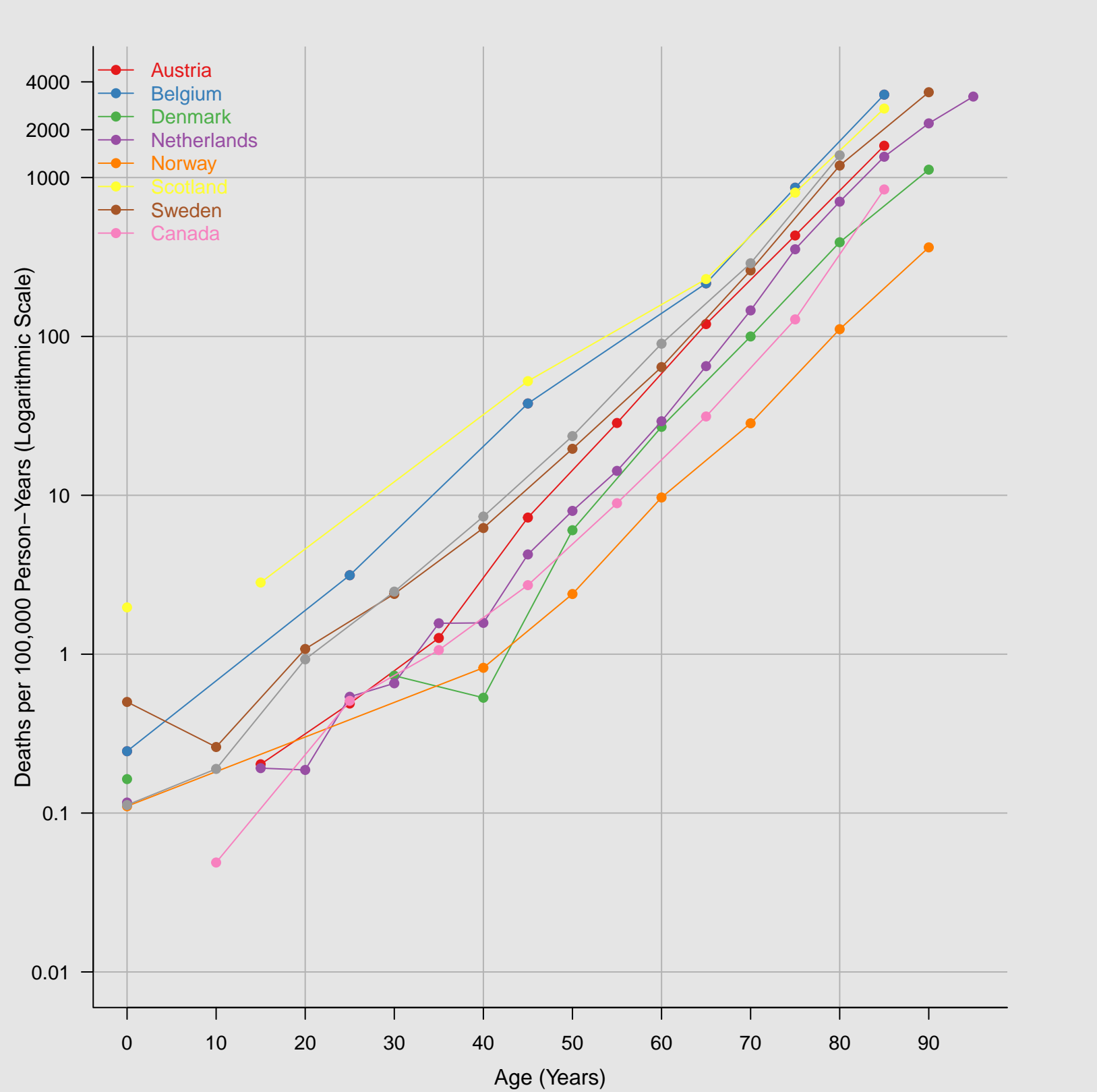

Supplement: Supplementary file 1 — Additional file 1: Figure 1. [file 41118_2021_115_MOESM1_ESM.pdf]
